# Enhancement of Frequency-Specific Hemodynamic Power and Functional Connectivity by Transcranial Photobiomodulation in Healthy Humans

**DOI:** 10.3389/fnins.2022.896502

**Published:** 2022-06-10

**Authors:** Nghi Cong Dung Truong, Xinlong Wang, Hashini Wanniarachchi, Hanli Liu

**Affiliations:** Department of Bioengineering, University of Texas at Arlington, Arlington, TX, United States

**Keywords:** transcranial photobiomodulation, functional near-infrared spectroscopy, functional connectivity, cortical infra-slow oscillation, endogenic oscillation, myogenic oscillation

## Abstract

Transcranial photobiomodulation (tPBM) has been considered a safe and effective brain stimulation modality being able to enhance cerebral oxygenation and neurocognitive function. To better understand the underlying neurophysiological effects of tPBM in the human brain, we utilized a 111-channel functional near infrared spectroscopy (fNIRS) system to map cerebral hemodynamic responses over the whole head to 8-min tPBM with 1,064-nm laser given on the forehead of 19 healthy participants. Instead of analyzing broad-frequency hemodynamic signals (0–0.2 Hz), we investigated frequency-specific effects of tPBM on three infra-slow oscillation (ISO) components consisting of endogenic, neurogenic, and myogenic vasomotions. Significant changes induced by tPBM in spectral power of oxygenated hemoglobin concentration (Δ[HbO]), functional connectivity (FC), and global network metrics at each of the three ISO frequency bands were identified and mapped topographically for frequency-specific comparisons. Our novel findings revealed that tPBM significantly increased endogenic Δ[HbO] powers over the right frontopolar area near the stimulation site. Also, we demonstrated that tPBM enabled significant enhancements of endogenic and myogenic FC across cortical regions as well as of several global network metrics. These findings were consistent with recent reports and met the expectation that myogenic oscillation is highly associated with endothelial activity, which is stimulated by tPBM-evoked nitric oxide (NO) release.

## 1. Introduction

Over the past decade, photobiomodulation (PBM) has gained considerable interest as an innovative modality for various disease treatments. This technique uses low-dose light from red to near-infrared (630–1,100 nm) to modulate tissue function. A large number of PBM studies on different conditions and diseases has shown positive outcome, including wound healing (Mester et al., 1971; Conlan et al., 1996; Yasukawa et al., 2007; Peplow et al., 2010), pain relief (Fulop et al., 2010), traumatic brain injury (Choi et al., 2012), Parkinson's disease (Quirk et al., 2012), and Alzheimer's disease (Grillo et al., 2013). Transcranial PBM (tPBM), which refers to PBM applied on the cerebral cortex, has also been demonstrated to enhance neural function and human cognition (Eells et al., 2004; Barrett and Gonzalez-Lima, 2013). tPBM mechanism has been suggested in relation to cytochrome c oxidase (CCO), a biological agent absorbing light in the NIR range (Chance et al., 1997; Karu, 1999). CCO is an essential enzyme associated with energy generation within the mitochondria and thus a biological mediator of PBM. Light absorption by CCO leads to an increase of CCO activity, release of nitric oxide (NO), and thus an increase of ATP production (Pastore et al., 1996; Hu et al., 2007). As a consequence, tPBM enhances cerebral blood flow (CBF) (Nawashiro et al., 2012; Lee et al., 2017) and brain energy metabolism (Rojas et al., 2012; Purushothuman et al., 2014).

Different imaging modalities have been utilized concurrently with tPBM administration to investigate its effects on the brain, such as electroencephalography (EEG) (Wang et al., 2019; Ghaderi et al., 2021), functional magnetic resonance imaging (fMRI) (Vargas et al., 2017; Dmochowski et al., 2020), broadband near-infrared spectroscopy (bbNIRS) (Tian et al., 2016; Wang et al., 2017b; Pruitt et al., 2020; Saucedo et al., 2021), or functional near-infrared spectroscopy (fNIRS) (Holmes et al., 2019; Urquhart et al., 2020b). Among them, NIRS (both fNIRS and bbNIRS) has been considered as an effective and non-invasive tool for assessing the hemodynamic (Villringer et al., 1993; Boas and Franceschini, 2011; Quaresima et al., 2012; Boas et al., 2014; Scholkmann et al., 2014) and metabolic state of the brain (Villringer et al., 1993; Smith, 2011; Tian et al., 2016; Wang et al., 2017b). The level of near-infrared light attenuation in tissue can be used to quantify the changes in chromophore concentrations of the investigation site and hence monitor tissue hemodynamic and metabolic state in real time. Both bbNIRS and fNIRS have advantages and disadvantages. The former is able to monitor changes in CCO metabolism but limited with the number of bbNIRS channels, especially for applications that require multi-channel monitoring. On the other hand, fNIRS can provide regional or global mapping of cerebral hemodynamics (Chen et al., 2020; Wanniarachchi et al., 2021), which helps better understand the influence of any intervention, including tPBM, on different cortical regions.

One primary source of cerebral activity is the vasomotion (Brown et al., 2002; Aalkjær and Nilsson, 2005; Vermeij et al., 2014; Bosch et al., 2017), which is associated to spontaneous oscillations derived from the blood vessel wall. There are three main intrinsic infra-slow oscillation (ISO) components of cerebral activity, including endogenic oscillation (0.003–0.02 Hz) relating to the vascular endothelial metabolic activity (Rubanyi, 1991), neurogenic oscillation (0.02–0.04 Hz) of the intrinsic neuronal activity, and myogenic oscillation (0.04–0.15 Hz) reflecting the vascular smooth muscle activity. ISO can be estimated using fNIRS, followed by appropriate frequency-domain analysis methods (Cao et al., 2018; Urquhart et al., 2020a). Analyzing cerebral ISO activities facilitates to better understand the neurophysiological responses under different stimulation conditions or diseases.

This work shared the data reported earlier (Urquhart et al., 2020b) that utilized a 111-channel fNIRS system concurrently with tPBM delivered on the right prefrontal cortex (rPFC) of 19 healthy participants to investigate the cerebral hemodynamic activity over the whole cortex. Compared to our previous work (Urquhart et al., 2020b), this paper reports several new analysis methodology and novel findings. First, the cluster-based permutation test (CBPT) (Maris and Oostenveld, 2007; Oostenveld et al., 2011; Pellegrino et al., 2016; Benavides-Varela and Gervain, 2017) was applied to the 111-channel fNIRS data to identify tPBM-evoked, brain-wide changes of oxygenated hemoglobin concentration (Δ[HbO]). Second, we analyzed frequency-specific hemodynamic signals by quantifying three ISO-based metrics under the active/sham tPBM conditions. The novel findings of this publication included (1) quantification of ISO powers of Δ[HbO], (2) ISO-specific functional connectivity (FC) across the whole cortex, and (3) global topographical network metrics at three ISO frequency bands. A few existing tPBM-related studies assessed FC computed from EEG (Zomorrodi et al., 2019; Ghaderi et al., 2021), fMRI (Dmochowski et al., 2020; Naeser et al., 2020), or broad-frequency fNIRS (Urquhart et al., 2020b), but not frequency-specific fNIRS. Therefore, the current work would help answer two key questions: Does tPBM modulate FC differently at different ISO frequencies? If so, which ISO frequency bands are more responsive to tPBM? We hypothesized that tPBM would enhance FC at specific frequency bands, depending on molecular mechanisms of tPBM. By the end of this work, our results would show significant increase in ISO powers of Δ[HbO], FC and global network metrics for endogenic or/and myogenic oscillations, which confirmed our hypothesis.

## 2. Materials and Methods

### 2.1. Participants

A total of 19 healthy adults (14 men, 5 women), mean age ± standard deviation (SD) of 31.7 ± 9.5 years, were recruited for this study. All participants were healthy and did not have any history of psychiatric or neurological disorders. The experimental protocol was approved by the Institutional Review Board (IRB) of the University of Texas at Arlington (IRB # 2017-0859). Informed consent was required prior to all experiments. Of the original 19 participants, two were excluded due to significant noise during the fNIRS acquisition, which left 17 subjects for further analysis.

### 2.2. Experimental Procedures

**Figure 1:**
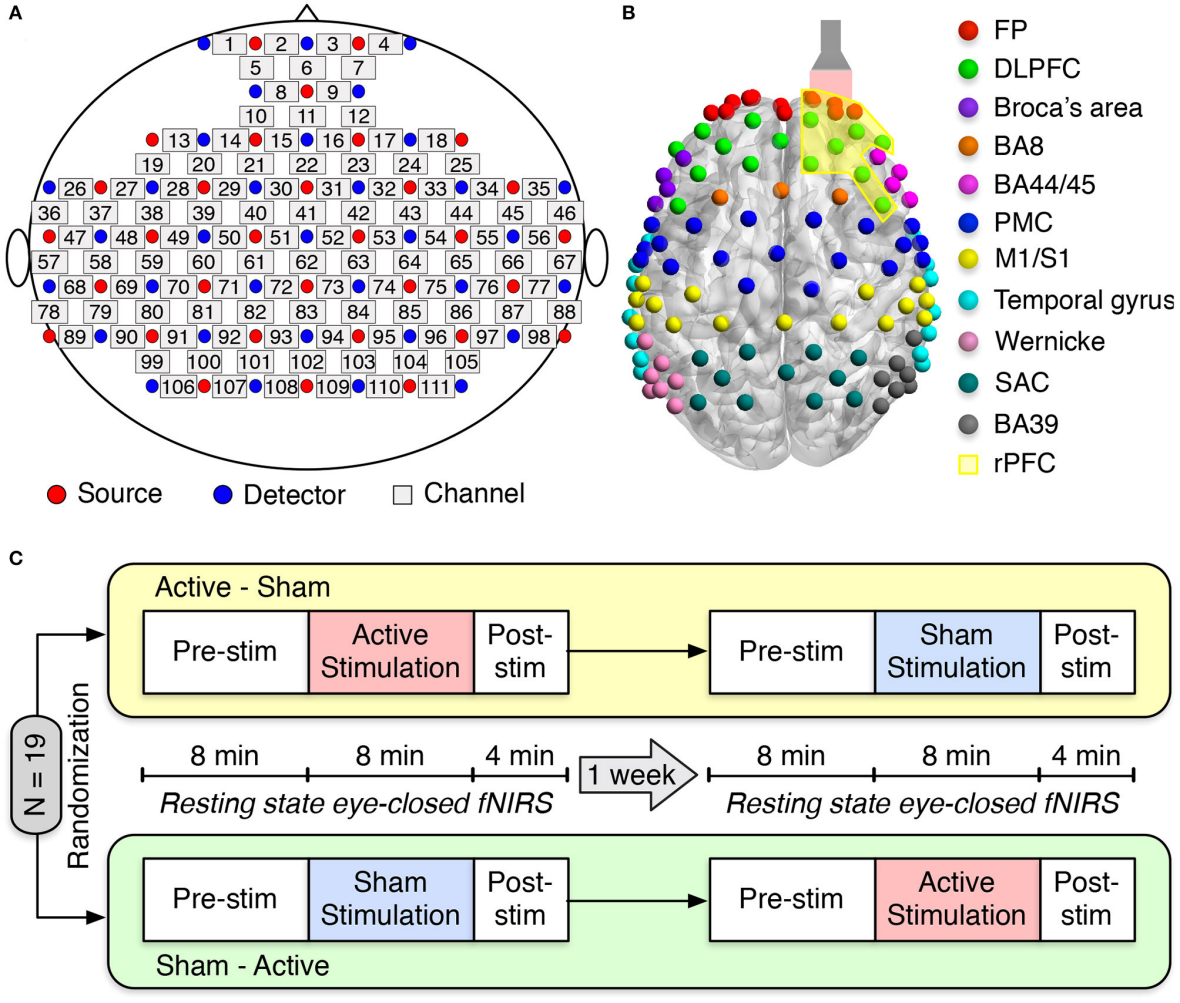
Experimental setup and protocol. **(A)** fNIRS source-detector configuration. The source and detector optodes are denoted as the red and blue circles, whereas squares represent the functional channels. **(B)** The distribution of 111 functional channels over different brain cortices: frontopolar area (FP) (red), dorsolateral prefrontal cortex (DLPFC) (green), Broca's area (purple), BA8 (orange), BA44/45 (pink), premotor cortex (PMC) (blue), primary motor and somatosensory cortices (M1/S1) (yellow), temporal gyrus (cyan), Wernicke's area (olive), somatosensory association cortex (SAC) (teal), and BA39 (gray). The flashlight represents the laser beam delivered to the right forehead of the subject. The yellow-shaded area corresponds to the right prefrontal cortex (rPFC). **(C)** Schematic diagram of the experimental protocol. Nineteen subjects were randomly divided into two groups: active-sham or sham-active stimulation. A minimum 1-week waiting period between two visits was required to avoid potential effects from active tPBM.

We employed a multichannel continuous-wave (CW) fNIRS system (LABNIRS OMM-3000, Shimadzu Corporation, Kyoto, Japan) with laser diodes at three near-infrared wavelengths (780, 805, and 830 nm) to measure cerebral hemodynamic responses. LABNIRS utilized Multialkali photomultiplier tubes as photodetectors, which have a very low quantum yield and have non-detectability at 1,064 nm used in our tPBM. Therefore, the interference from the tPBM laser to fNIRS measurements was minimal. Detected signals were sampled at 10.1 Hz. Thirty-two emitters and thirty-four detectors were arranged over the entire head, resulting in a 111-channel layout (Figure 1A). Anatomical optode locations with respect to the standard cranial reference points (nasion, inion, left and right preauricular) were determined for each subject using a 3D digitizer (FASTRAK, Polhemus VT, USA). The Montreal Neurological Institute (MNI) coordinates and the corresponding Brodmann Area (BA) for each channel were determined using the NIRS-SPM software (Ye et al., 2009). One hundred and eleven fNIRS channels covered eleven main cortical areas as shown in Figure 1B: frontopolar area (FP) (red), dorsolateral prefrontal cortex (DLPFC) (green), Broca's area (purple), BA8 (orange), BA44/45 (pink), premotor cortex (PMC) (blue), primary motor and somatosensory cortices (M1/S1) (yellow), temporal gyrus (cyan), Wernicke's area (olive), somatosensory association cortex (SAC) (teal), and BA39 (gray).

An FDA-cleared 1,064-nm CW laser (Model CG-5000 Laser, Cell Gen Therapeutics LLC, Dallas, Texas) was used for non-invasive tPBM (Barrett and Gonzalez-Lima, 2013; Tian et al., 2016; Wang et al., 2016, 2017b, 2019; Blanco et al., 2017). The laser's irradiation area was 13.6 cm^2^, and the laser power was set to 3.4 W. This laser power resulted in a power density of 0.25 W/cm^2^ and a total energy dose of 1,632 J over 8-min tPBM duration (3.4 W × 60 s/min × 8 min = 1,632 J). The light was applied by non-contact delivery over the right frontopolar area near the Fp2 location according to the 10/20 international standard system (Jurcak et al., 2007) (Figure 1B). For sham stimulation, the laser power was set to 0.1 W, and a black cap was placed in front of the laser aperture to further block the light. The reason to keep 0.1 W power was to keep all the electronic components on while the light delivery was zero. The participants would not be aware of the cap since it was put after they closed their eyes. Experimental participants must wear a pair of laser protection goggles throughout the experiment.

Each subject attended two study sessions: active tPBM and sham tPBM. The orders of these two sessions were randomized among nineteen subjects. A minimum 1-week waiting period between two visits was required to avoid any carry-over effects. During the experiment, subjects were instructed to sit comfortably with their eyes closed. The resting-state fNIRS data were recorded for 8 min of pre-stimulation, 8 min of active/sham stimulation, and 4 min of post-stimulation (Figure 1C).

### 2.3. Data Preprocessing

**Figure 2:**
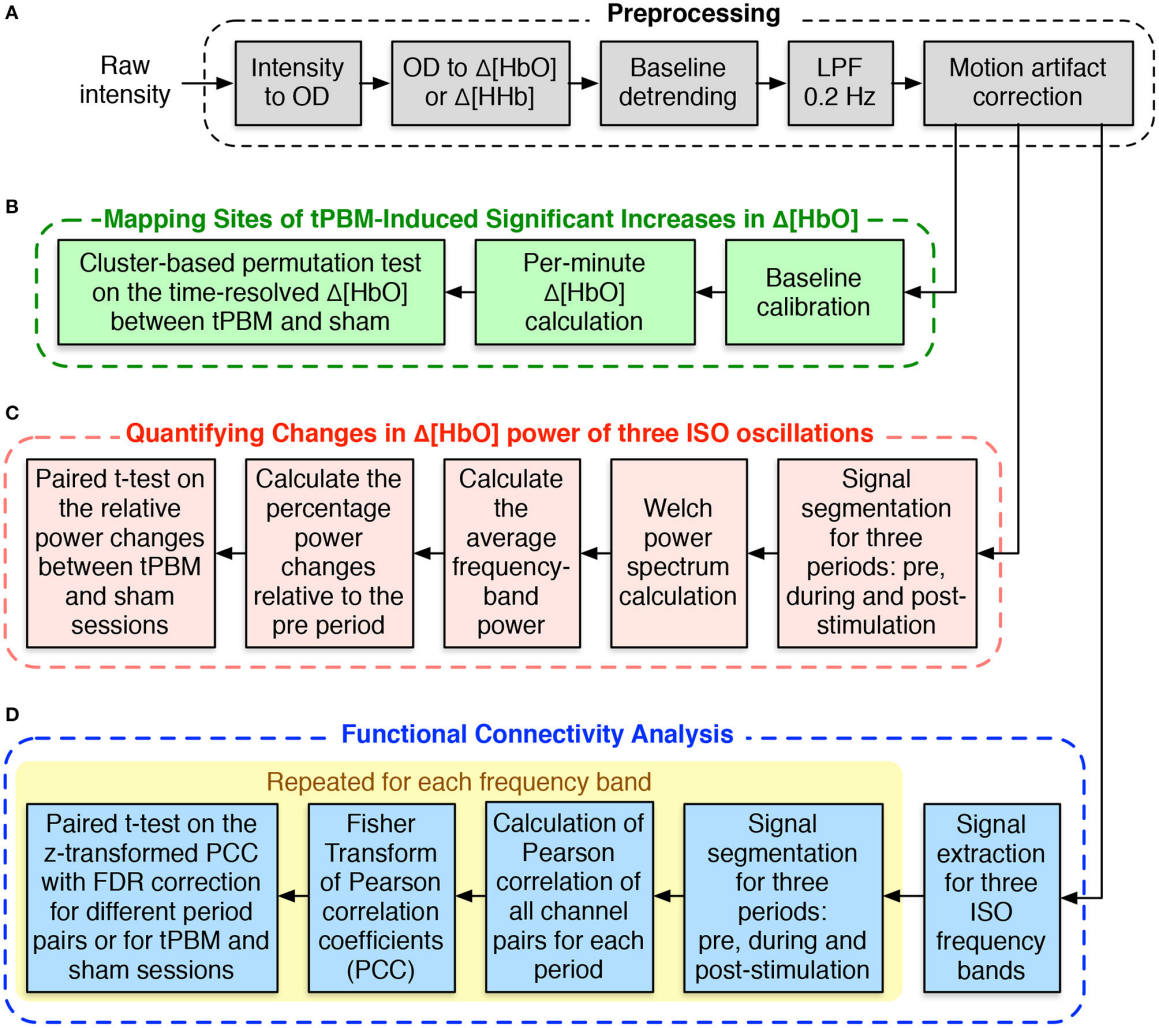
Flowchart for fNIRS data analysis. **(A)** fNIRS data preprocessing procedure. **(B)** Processing steps for mapping sites of tPBM-induced significant increases of Δ[HbO]. **(C)** Processing procedure for quantifying changes in Δ[HbO] power of three ISOs. **(D)** Steps for functional connectivity analysis.

Figure 2A depicts different steps of preprocessing the resting-state fNIRS data collected in our study. We followed the typical fNIRS data pre-processing pipeline to process our resting-state fNIRS data (Pinti et al., 2019; Hou et al., 2021). The raw voltage data were first converted into optical density data and then into the concentration changes of oxy-hemoglobin (Δ[HbO]) and deoxy-hemoglobin (Δ[HHb]) using the modified Beer-Lambert law (Liu et al., 2000; Sassaroli and Fantini, 2004) with a differential pathlength factor of 6. These two steps were done with the functions from the open-source Homer2 software package (Huppert et al., 2009). Since Δ[HbO]) and Δ[HHb] may suffer from the systematic drifts due to instrument instability, the linear trend was first estimated using 8-min baseline signals and then subtracted from the whole signals. Subsequently, Δ[HbO] and Δ[HHb] were low-pass filtered using a third-order Butterworth filter with a cut-off frequency of 0.2 Hz to remove physiological noise, such as respiration (0.2–0.5 Hz), heartbeat (1–15 Hz), and other instrument noise (Pinti et al., 2019). Finally, motion correction using Principle Component Analysis (PCA) filter (Zhang et al., 2005) was applied to remove motion artifacts. The first two principal components, accounting for the most significant proportion of the variance in the data, were removed from the signals of all channels. Finally, we applied the signal quality index (SQI) algorithm (Sappia et al., 2020) to evaluate the quality of the fNIRS signals. Channels whose SQI was equal to 1, indicating a very low-quality signal, were marked as excluded and would not be considered in the statistical analysis. Of the original 19 participants, two whose fNIRS data had more than 10 rejected channels were excluded, leaving 17 subjects for further analysis. Due to smaller amplitudes and similar inverse patterns of Δ[HHb], we considered only Δ[HbO] for further data analysis.

### 2.4. Data Analysis

#### 2.4.1. Mapping Sites of tPBM-Induced Significant Increases in Δ[HbO]

As mentioned in Section 1, we first considered the whole-head, broad-frequency Δ[HbO] signals and sought to identify cerebral regions responding significantly to tPBM. We applied the CBPT (Maris and Oostenveld, 2007; Oostenveld et al., 2011; Pellegrino et al., 2016; Benavides-Varela and Gervain, 2017) to the whole-head time-resolved Δ[HbO] signals of tPBM and sham sessions. Such an analysis allows us to identify significant spatio-temporal clusters exhibiting hemodynamic responses to tPBM sustained in time and spatial neighborhood channels. Also, CBPT helps avoid the statistical problem of overly strict correction for multiple comparisons, which multi-channel fNIRS data often encounter.

The principle of permutation-based statistical testing is that observations for different conditions in the null hypothesis are drawn from the same distribution and thus exchangeable. Therefore, if an effect observed in the data is not spurious, it should not be perceived when the data is randomly permuted multiple times. If less than 5% of the permutations show the effect, the null hypothesis is rejected, and the observed effect is considered genuine and thus significant (Maris and Oostenveld, 2007). The CBPT further considers both spatial and temporal adjacency of the observed data based on the property that observations on neighboring sites and time points are usually correlated. Once cluster candidates are formed, the cluster-level *t*-values, estimated as the sum of *t*-values of all data points belonging to each cluster, are compared to the permutation distribution of maximal cluster statistics. Such a permutation distribution is generated by randomly permuting the data a large number of times and retrieving the maximum cluster statistics (i.e., the maximum of the cluster-level summed *t*-values) every time. Any clusters whose cluster-level *t*-values from the actual data are larger than the 95th percentile of the maximum cluster permutation distribution are considered significant (Oostenveld et al., 2011; Pellegrino et al., 2016).

The processing routine to perform the CBPT-based Δ[HbO] time-series data analysis is shown in Figure 2B. First, we calculated the average of Δ[HbO] over 1 min prior to the active/sham stimulation and subtracted it from the corresponding Δ[HbO] time-series. The baseline-calibrated Δ[HbO] signals during 8 min of stimulation and 4 min of post-stimulation were then averaged or segmented for every 1 min. CBPT was finally performed on the time-dependent per-minute Δ[HbO] values of all 111 channels using the functions from the Fieldtrip toolbox (Oostenveld et al., 2011). The channel neighbors for spatial clustering were defined within a distance of 2.5 cm from the center channel, which resulted in an average of 4.7 neighbors per channel. The critical cluster threshold for considering a data point (a channel-time pair in our case) as a candidate member of a cluster was computed based on the statistical distribution of the permutation data and the cluster alpha of 0.05. The number of randomizations or permutations was set to 2,000. The Monte Carlo method was used to calculate the probability of each cluster. Clusters whose *p*-values were less than the critical α_*cluster*_ = 0.05 were considered significant.

#### 2.4.2. Quantification of Changes in Δ[HbO] Power of Three Infra-Slow Oscillations (ISO)

The data analysis on CBPT-based Δ[HbO] time-series given in the previous section only investigated the broad-frequency (0–0.2 Hz) hemodynamic changes without considering frequency-specific oscillations. Our interest in this section is to quantify the changes in Δ[HbO] power at three ISO frequency bands, including endogenic (0.003–0.02 Hz), neurogenic (0.02–0.04 Hz), and myogenic (0.04–0.15 Hz) oscillations. Welch method (Welch, 1967) was used to calculate the power spectral density (PSD) for Δ[HbO] time-series of significant channels (channels 3, 4, and 7) found in Section 2.4.1. The processing procedure is shown in Figure 2C. First, we segmented Δ[HbO] signals based on three experimental periods, namely, 8 min of baseline (*pre*), 8 min of tPBM/sham (*stim*), and 4 min of post-tPBM/sham (*post*). Let $P_{<seg>}^{c}\left(f\right)$ be the Welch power spectrum of Δ[HbO] segment named <*seg*> from channel *c*, where <*seg*> was noted as “*pre*”, “*stim*”, or “*post*”. The average power of each ISO frequency band $P_{<seg>,<fb>}^{c}$ was then computed by averaging the frequency-dependent power within the corresponding frequency range of the ISO frequency band <*fb*>, including “*e*” for endogenic, “*n*” for neurogenic, and “*m*” for myogenic. Finally, the percentage changes of the power during tPBM/sham and post-tPBM/sham periods to the power during the baseline period were defined as follows:


(1) $$\begin{array}{l} \Delta P_{<seg>,<fb>}^{c}=\frac{P_{<seg>,<fb>}^{c}-P_{pre,<fb>}^{c}}{P_{pre,<fb>}^{c}}\times 100\% \end{array}$$

The percentage changes in power relative to the baseline power were calculated for both tPBM and sham sessions. Paired *t*-test was finally employed to investigate the effects of tPBM on the relative changes in Δ[HbO] power of three ISO frequency bands.

#### 2.4.3. Quantification of Changes in Functional Connectivity of Three ISO Frequency Bands

We desired to investigate further the effect of tPBM on brain connectivity by calculating FC for each experimental period: pre-stimulation, during stimulation, and post-stimulation for both study sessions (active and sham tPBM). Unlike our previously published work (Urquhart et al., 2020b) that computed FC using Δ[HbO] signals from the entire range of hemodynamic fluctuations (0–0.2 Hz), we calculated FC for three ISO frequency bands. The pipeline of the FC analysis is thus depicted in Figure 2D. First, the broadband Δ[HbO] time series was first bandpass filtered to extract the signals corresponding to three ISO frequency bands. The time-series data of each ISO were then segmented for three periods, corresponding to the baseline (*pre*), during tPBM/sham (*stim*), and post-tPBM/sham (*post*). Pearson's correlation coefficients (PCC) were calculated for each pair of 111 fNIRS channels (Niu and He, 2014). The PCC matrices were finally converted to *z*-values using Fisher's r-to-z transformation to ensure normality.

Since the tPBM stimulation site was near the right prefrontal cortex (rPFC), only FCs between channels within the rPFC (yellow shaded region shown in Figure 1B) to all other channels were considered for further statistical analysis. We were interested in changes in functional connectivity in both cases: (1) over time (pre vs. during vs. post) within each experimental session (active tPBM or sham) and (2) between experimental sessions (tPBM vs. sham). To investigate the effects of tPBM on brain connectivity over time, we compared the z-transformed PCC between each period pair: stimulation vs. pre, post vs. stimulation, post vs. pre. Paired *t*-test was used to compare the z-transformed PCC of two periods across subjects within the same experimental session (tPBM or sham). False discovery rate (FDR) correction with q-value = 0.05 was employed for multiple comparison correction. This procedure was applied for both tPBM and sham sessions to ensure the significant differences between any period pair were genuinely provoked by tPBM.

For the between experimental sessions comparison (tPBM vs. sham), paired *t*-tests with FDR correction was also employed to compare the z-transformed PCC between tPBM and sham experiments of each experimental period. This procedure was repeated for all three periods (pre, during, and post-stimulation) and for three ISO frequency bands.

#### 2.4.4. Topographical Network Metrics Analysis for Three ISO Frequency Bands

Graph theory analysis (GTA) (Niu et al., 2012, 2013; Li et al., 2018) was also employed to investigate tPBM-induced changes in global topographical network metrics for three ISO frequency bands. For each frequency-specific FC matrix obtained in Section 2.4.3, we constructed the neighborhood graph in which fNIRS channels were considered as nodes and FC between two channels were considered as edges. The neighborhood graph was then converted into an adjacency matrix by thresholding it with different sparsity levels *S* (0.15 < *S* < 0.5) to obtain binary networks. Seven global network metrics, including small-world properties (clustering coefficient *C*_*p*_, characteristic path length *L*_*p*_, normalized clustering coefficient γ, normalized characteristic path length λ, and small-world σ) and efficiency parameters (local efficiency *E*_*loc*_ and global efficiency *E*_*g*_), were calculated from the binary networks of three periods (pre, during tPBM/sham, and post) of both study sessions (active and sham tPBM). The global network metrics were finally compared between active and sham tPBM sessions using the relative changes to the baseline period (i.e., pre-tPBM/sham). The relative change of a given network metric was calculated as:


(2) $$\begin{array}{l} \Delta M_{<p>,<fb>}=\frac{M_{<p>,<fb>}-M_{pre,<fb>}}{M_{pre,<fb>}}\times 100\% \end{array}$$

where *M*_<*p*>,<*fb*>_ denotes the network metric *M* obtained from the FC of period <*p*> at ISO frequency band <*fb*>.

## 3. Results

### 3.1. Mapping Sites of tPBM-Induced Significant Increases in Δ[HbO] Using CBPT

**Figure 3:**
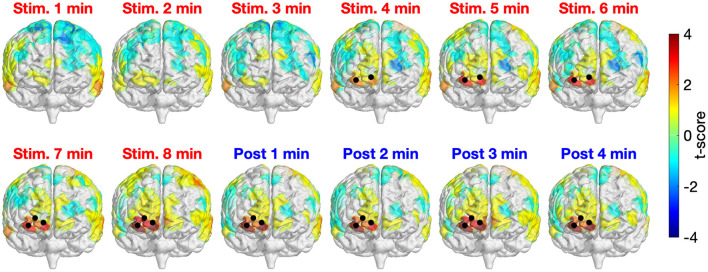
Time-resolved topographical t-maps and CBPT results when comparing the whole head Δ[HbO] time series of two study sessions (active and sham tPBM). Black dots indicate the channels that contributed to significant clusters obtained by the CBPT (*p*_*cluster*_ < 0.05).

Figure 3 depicts the time-resolved (in minute) topographical t-maps and CBPT results when comparing the whole head Δ[HbO] time series of two study sessions (active and sham tPBM). The positive values in the topographical t-map indicate that the Δ[HbO] values of the active tPBM session were higher than those of the sham tPBM session, whereas the negative values denote the opposite. Black dots represent the channels that contributed to significant clusters obtained by the CBPT (*p*_*cluster*_ = 0.05).

For the first 3 min after starting the stimulation, no significant differences between active and sham tPBM were detected. Starting from minute 4, CBPT revealed significant differences between active and sham tPBM over the right frontopolar area (channels 3, 4, and then channel 7 starting from minute 7). It is worth noting that the topographical t-maps also show the gradual increase of the t-score values of the channels near the stimulation site (the surface color gradually changed from yellow to orange to bright red and then dark red). Moreover, the increase of Δ[HbO] remained during the 4-min post-stimulation when the stimulation light was off. Therefore, such an increase in Δ[HbO] is expected to truly come from the physiological responses to tPBM. The contamination effect from the laser light would have shown an immediate reduction in Δ[HbO] after the laser ceased.

**Figure 4:**
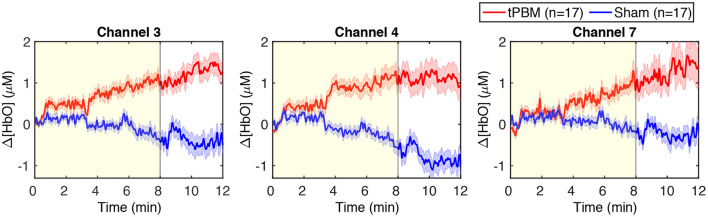
Time-resolved Δ[HbO] signals of three channels near the stimulation site: channels 3, 4, and 7. The red and blue curves correspond to the tPBM and sham sessions, respectively. Red and blue shades indicate the standard error of the mean of each group. The yellow shade depicts the stimulation period (8 min).

We also present in Figure 4 the time-resolved Δ[HbO] signals of three channels near the stimulation site to better visualize the changes in HbO concentration induced by tPBM. We can notice a gradual increase of Δ[HbO] under the tPBM condition (red curves). As expected, notable differences of Δ[HbO] curves between active and sham tPBM were observed from minute 4 of stimulation. Moreover, the increase of Δ[HbO] remains during the 4-min post-stimulation (minutes 8–12) when the stimulation was off.

### 3.2. Changes in Δ[HbO] Power of Three ISO Frequency Bands

**Figure 5:**
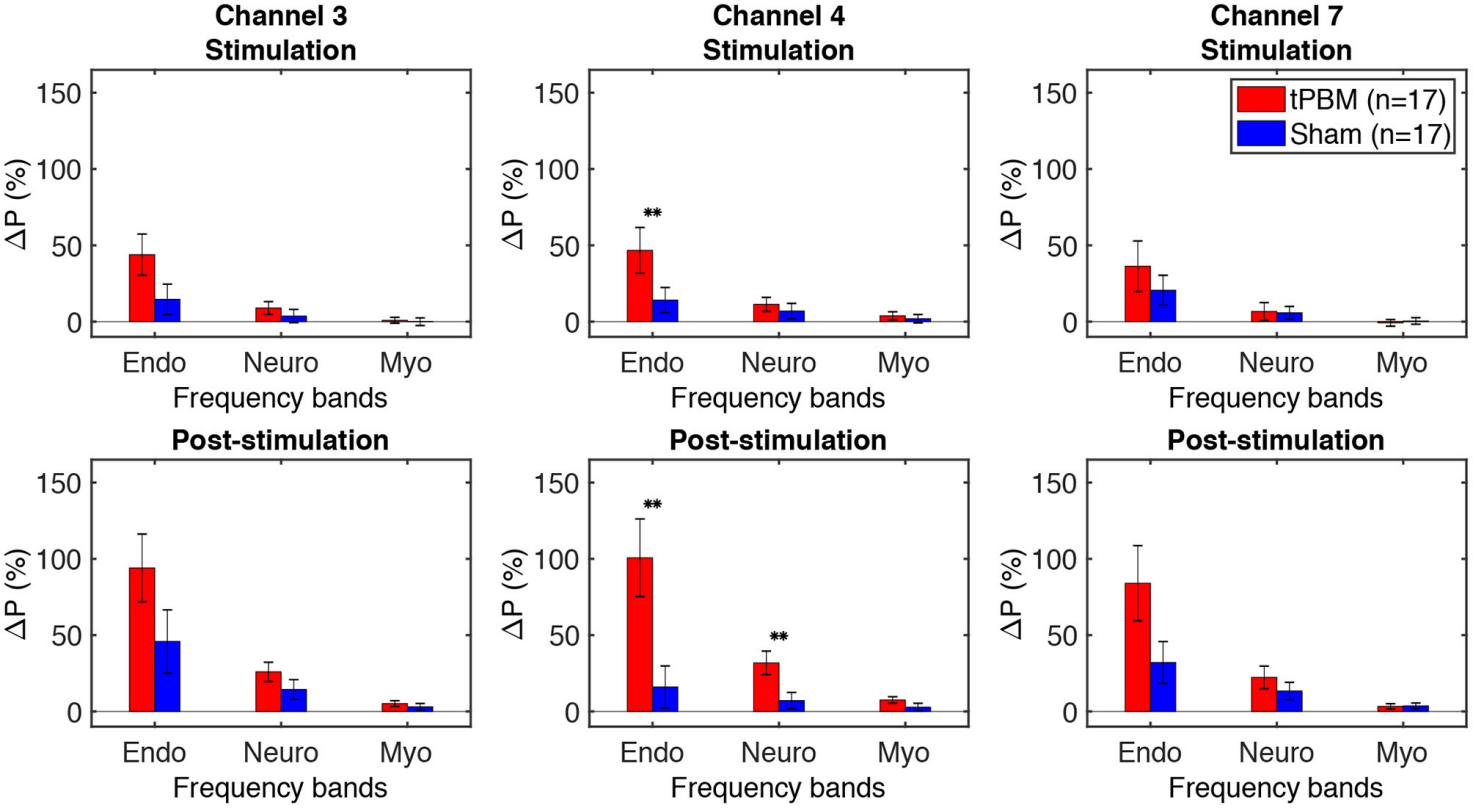
Percentage changes of Δ[HbO] power Δ*P* of three significant channels (3, 4, and 7) for the tPBM/sham stimulation (first row) and post-stimulation (second row) periods. tPBM- and sham-induced Δ*P* are marked by red and blue bars, respectively. Error bars indicate the standard error of the mean of each group. Statistical results obtained by paired *t*-tests between tPBM and sham sessions are marked as “**” for *p* < 0.01.

To assess tPBM-induced effects on hemodynamic power of three ISO frequency bands, we computed the percentage power changes for both tPBM and sham sessions based on Eq. (1). Figure 5 depicts the percentage changes of hemodynamic power Δ*P* of three significant channels detected in Section 3.1 for the tPBM/sham stimulation period and the post-tPBM/sham period. Statistical results obtained by paired *t*-tests between tPBM and sham sessions are marked as “**” for *p* < 0.01. Compared to sham condition, tPBM significantly enhanced Δ*P* of channel 4 in the endogenic frequency band for both tPBM and post-tPBM periods. Significant increase in relative power changes was also revealed in channel 4 in the neurogenic frequency band during the post-tPBM period.

### 3.3. Changes in Functional Connectivity of Three ISO Frequency Bands

#### 3.3.1. Channel-Wise Analysis of Frequency-Specific FC

**Figure 6:**
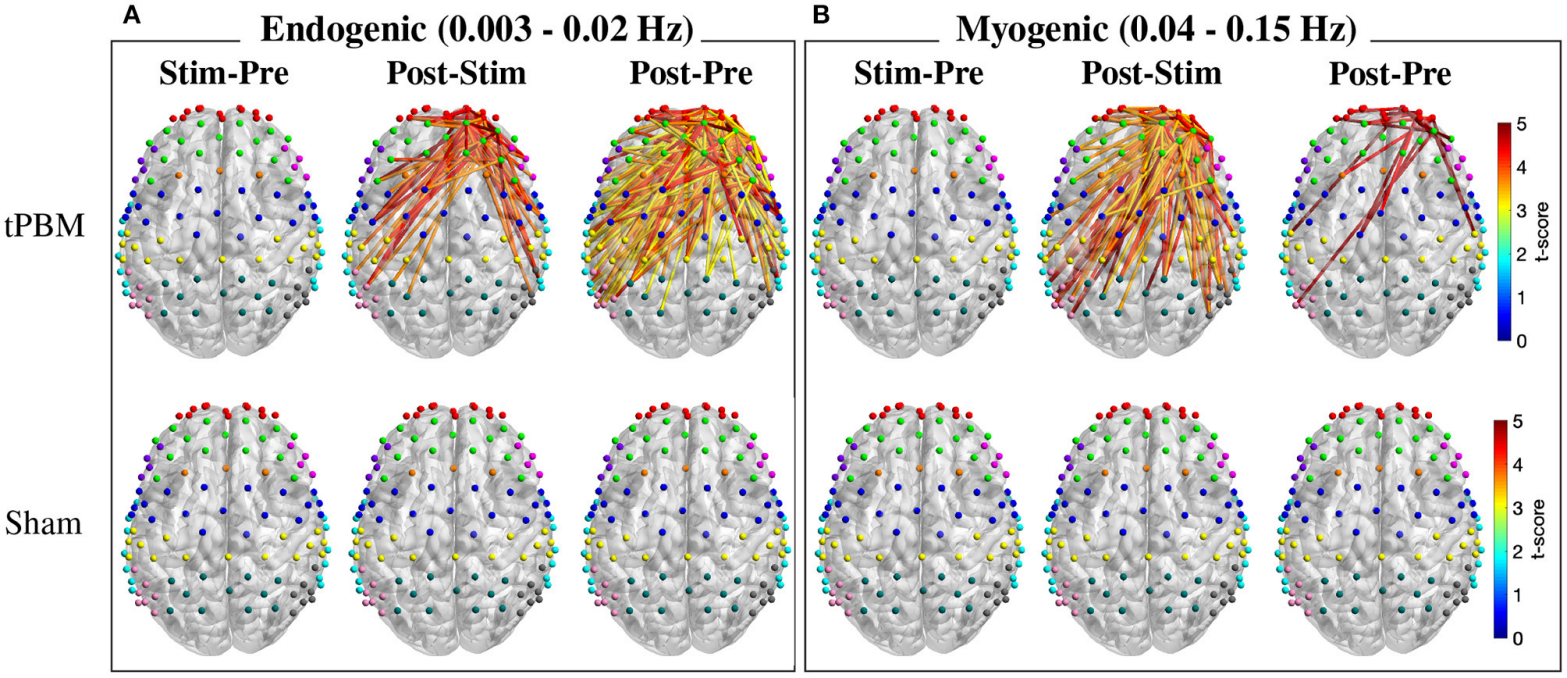
T-score maps of significant changes in FC between three pairs of periods for two frequency bands: **(A)** endogenic and **(B)** myogenic (*p* < 0.05, FDR corrected). Only FC from right PFC to all channels was considered for the statistical analysis. The FC strength was compared between stimulation vs. pre-stimulation (Stim-Pre), post- vs. during stimulation (Post-Stim), and post- vs. pre-stimulation (Post-Pre) for both active (first row) and sham (second row) tPBM.

As mentioned in Section 2.4.3, FC was calculated for three ISO frequency bands. Paired *t*-tests with FDR correction were first employed to determine significant FC between three pairs of periods, namely, Stim-Pre (during vs. pre-stimulation), Post-Stim (post vs. during stimulation), and Post-Pre (post vs. pre-stimulation) within the same experiment session (active and sham tPBM). Figure 6 depicts the t-score maps of FC between three pairs of periods for the endogenic and myogenic bands. For the endogenic frequency band (Figure 6A), significant differences in FC were found in the case of active tPBM for the Post-Stim (first row, second column) and Post-Pre stim (first row, third column) pairs (*p* < 0.05, FDR corrected). The connections indicated the stronger FC during post-stimulation than pre or during stimulation. No significant differences in FC were observed for the Stim-Pre pair of the active tPBM experiment (first row, first column). Also, paired *t*-tests with FDR correction did not reveal any significant FC for all three pairs of periods for the sham tPBM case (second row), which confirmed the genuine effects induced by active tPBM.

Specifically, for the Post-Pre pair of the active tPBM experiment (Figure 6A), a large number of significant connections (*p* < 0.05, FDR corrected) were observed among channels within the rFP or between rFP and rDLPFC (10 significant connections among channels within the rFP and 8 between rFP and rDLPFC). Other ROI pairs having a high number of significant connections included rDLPFC and lM1/S1 (10), rFP and lDLPFC (7), rDLPFC and rPMC (7), rFP and lPMC (6), rFP and lM1/S1 (5), rFP and BA44/45 (5), rDLPFC and lDLPFC (5), rDLPFC and rDLPFC (6), rDLPFC and lPMC (6), and rDLPFC and Wernicke (5). Thus, in addition to the two regions FP and DLPFC located close to the excitation region, other ROIs including PMC, M1/S1, and Wernicke also showed significant improvements in FC between them and the rPFC.

Meanwhile, the myogenic band exhibited more and stronger FC for the Post-Stim period pair than Stim-Pre and Post-Pre pairs for the case of active tPBM (first row of Figure 6B). Many significant connections were found when comparing FC between Post-Stim. FC during post-stimulation was also enhanced compared with the pre-stimulation phase. Once again, no significant FC was found for the case of sham tPBM for all three pairs of periods (second row of Figure 6B).

For the neurogenic band, statistical analysis of FC between three pairs of periods could not reveal any significant changes in connections for both active and sham tPBM experiments. Thus, no report for this frequency band was presented.

Regarding the comparison between tPBM and sham sessions, no significant difference in FC was revealed after paired *t*-tests with FDR correction for any experimental period (pre, during, or post-stimulation) for all three ISO frequency bands. Thus, we did not report any results for this comparison.

#### 3.3.2. Region-Wise Analysis of Frequency-Specific FC

**Figure 7:**
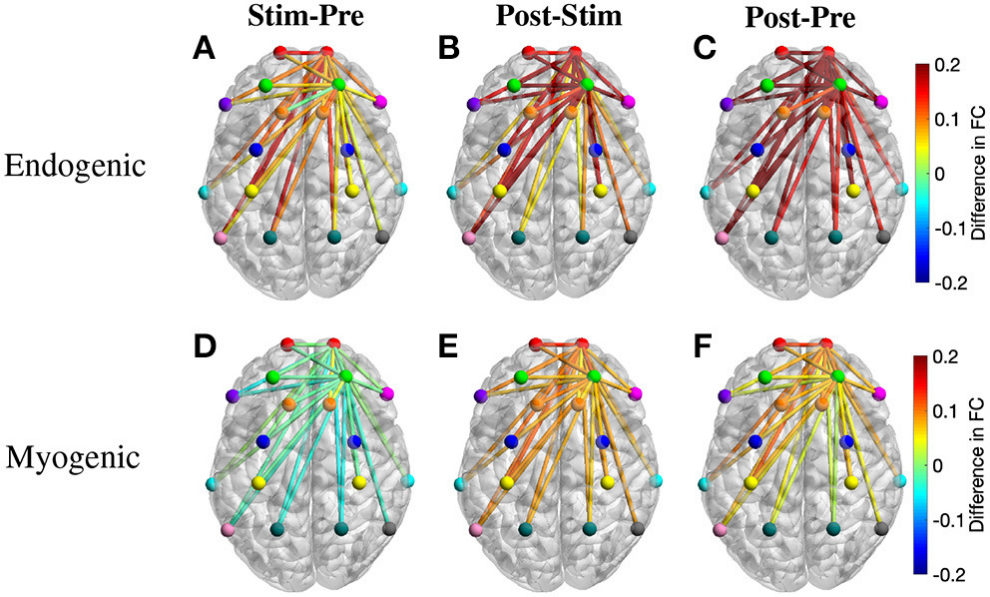
Topographical maps of the region-wise difference in FC between three pairs of periods for two frequency bands: **(A–C)** endogenic and **(D–F)** myogenic. ROIs include left and right frontopolar area (lFP; rFP) (red), left and right dorsolateral prefrontal cortex (lDLPFC; rDLPFC) (green), Broca's area (purple), left and right BA8 (lBA8; rBA8) (orange), BA44/45 (pink), left and right premotor cortex (lPMC; rPMC) (blue), left and right primary motor and somatosensory cortices (lM1/S1; rM1/S1) (yellow), left and right temporal gyrus (lTemporal; rTemporal) (cyan), Wernicke's area (olive), left and right somatosensory association cortex (lSAC; rSAC) (teal), and BA39 (gray). Only FC from (lFP; rFP) and (lDLPFC; rDLPFC) to all ROIs was plotted.

To further understand the changes of FC over different periods, we also calculated the region-wise difference in FC of three period pairs of the active tPBM experiment. The average FC of all connections between two ROIs was first calculated, and the difference in region-wise FC between two periods, including Stim-Pre, Post-Stim, and Post-Pre, was then computed. Topographical maps of the region-wise difference in FC at the endogenic frequency band were depicted in Figures 7A–C, exhibiting a gradual enhancement of FC starting from the stimulation phase to the post-stimulation phase compared to pre-stimulation. FC during the stimulation period improved slightly compared to pre-stimulation (Figure 7A), which explains why paired *t*-tests with FDR correction did not reveal any significant connection when comparing these two periods (Figure 6A). FC during post-stimulation compared to the stimulation phase was enhanced more than the Stim-Pre period pair, which resulted in several significant connections after applying paired *t*-tests with FDR correction (Figure 6A). The region-wise difference in FC for this period pair (Figure 7B) also shows higher values than the Stim-Pre pair. In consequence, we observed higher difference in FC when comparing post and pre-stimulation periods (Figure 7C), as well as much more significant connections after paired *t*-tests with FDR correction as shown in Figure 6A.

The region-wise topographical maps of the difference in FC at the myogenic frequency band exhibited a slight decline in whole-head FC during the stimulation period compared to the pre-stimulation (Figure 7D). However, such a decline was not significant (i.e., no significant connection was found in Figure 6B). During the post-stimulation, global FC was enhanced notably, resulting in a large number of significant connections between channels from rPFC to all other channels observed for the Post-Stim pair of periods. Overall, FC was also improved after tPBM (the Post-Pre pair, Figure 6B).

### 3.4. Changes in Global Topographical Network Metrics for Three ISO Frequency Bands

As mentioned in Section 2.4.4 the relative changes of seven global network metrics were calculated for each ISO frequency band and for during and post-stimulation periods. Then, paired *t*-tests were used to compare the relative changes of network metrics between tPBM and sham conditions. In this section, we report only the GTA results revealed to be significantly different between these two experimental conditions.

**Figure 8:**
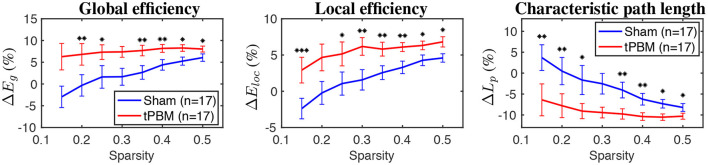
The relative changes of three global topological metrics during the stimulation period for the myogenic frequency band (from **left** to **right**: global efficiency, local efficiency, and characteristic path length). Significant differences between tPBM and sham conditions are marked by black stars: “*” for *p* < 0.05, “**” for *p* < 0.01, and “***” for *p* < 0.001. Red lines indicate tPBM condition, while blue lines indicate sham. Error bars indicate the standard error of the mean of each case.

Figure 8 shows relative changes of three global topological metrics, namely, global efficiency, local efficiency, and characteristic path length, during the stimulation period at the myogenic frequency band. Briefly, global efficiency (*E*_*g*_) depicts how information propagates efficiently through the whole network, while local efficiency (*E*_*loc*_) measures the average efficiency of information transfer within a node's neighborhood (Bullmore and Sporns, 2009). Finally, the characteristic path length corresponds to the average shortest path length between all pairs of nodes in the graph (Rubinov and Sporns, 2010).

GTA results showed that global and local efficiency were improved thanks to tPBM stimulation. Meanwhile, the characteristic path lengths during tPBM stimulation were significantly shortened than those under the sham treatment. Shorter path lengths indicate that tPBM enhanced network information exchange. As a consequence, both global and local efficiency of the topological network was improved due to the shorter characteristic path length induced by tPBM.

No significant differences were found during the post tPBM/sham period at the myogenic frequency band, as well as during both periods (i.e., during and post tPBM/sham) at endogenic and neurogenic bands. Thus, no report for other periods at other frequency bands was presented.

## 4. Discussion

### 4.1. tPBM-Induced Increases of Cerebral Δ[HbO] Near the Stimulation Site

In our previously published work (Urquhart et al., 2020b), we considered only eight channels in the prefrontal region and utilized the paired *t*-test at each of the eight channels without correction for multiple comparisons in statistical analysis. In this study, we re-analyzed the signals of all 111 channels and applied a more rigorous statistical analysis, CBPT, to investigate crucial channels/regions possessing significant increases of Δ[HbO] induced by tPBM. This approach revealed significant clusters over the right frontopolar area near the stimulation site. The positive t-score values of the channels contributing to those clusters denoted a substantial increase of Δ[HbO] values during the active tPBM session compared to those of the sham session. Moreover, notable increases in Δ[HbO] occurred 4 min after starting the laser stimulation and remained through the stimulation and post stimulation.

These findings were consistent with those reported previously (Tian et al., 2016; Wang et al., 2017b; Hipskind et al., 2018; Holmes et al., 2019; Pruitt et al., 2020). For example, in Wang et al. (2017b), 1,064-nm laser light was delivered to the forehead of healthy subjects and measured with a single-channel bbNIRS system to investigate corresponding changes in Δ[CCO], Δ[HbO], and Δ[HbT]. The authors observed significant increases in Δ[HbO] and Δ[CCO] near the stimulation location. Pruitt et al. (2020) repeated the same experimental protocol with two age groups of young (mean of 26.7 years of age) and older subjects (mean of 68.2 years of age). Experimental results also revealed similar net increases in sham-controlled Δ[HbO]. Another study evidenced the augment of Δ[HbO] over the frontal area before and after forehead tPBM measured with a 20-channel fNIRS system (Holmes et al., 2019). In the meantime, the authors also reported tPBM-evoked improvement of cognitive performance.

It is worth noting that, although the skin temperature at the local stimulation site was slightly increased because of local light absorption (Wang et al., 2017a; Pruitt et al., 2022), the increase of Δ[HbO] observed in this study was not caused by the light-induced thermal effect. As reported in the previous study from our group (Wang et al., 2017a), the changes in hemoglobin concentration induced by the heat stimulation were completely different from those provoked by tPBM. Specifically, thermal stimulation led to a decrease in Δ[HbO] during the stimulation period and a back-to-baseline state within 2 or 3 min during the post-stimulation period. Other investigations on the tPBM-induced increase in brain temperature through a computational model (Bhattacharya and Dutta, 2019) or magnetic resonance thermometry (Dmochowski et al., 2020) also confirmed that there was no significant difference in temperature between tPBM and sham conditions. Furthermore, a recent study using EEG to investigate the effects of tPBM and thermal stimulation demonstrated that alterations of EEG power topography were significantly different between the two stimulations (Wang et al., 2021).

### 4.2. tPBM-Induced Increases in Δ[HbO] Power at Endogenic Oscillation

To the best of our knowledge, all tPBM-related studies utilized only time-domain or time-averaged analysis to investigate significant changes in hemodynamic and metabolic signals induced by tPBM (Tian et al., 2016; Wang et al., 2017b; Hipskind et al., 2018; Holmes et al., 2019; Pruitt et al., 2020; Urquhart et al., 2020b). In this study, we employed a frequency-domain approach by taking the Welch method to investigate or analyze frequency-specific hemodynamic signals. The comparison of changes in frequency-dependent Δ[HbO] power between active tPBM and sham sessions revealed significant increase of Δ[HbO] power in the endogenic frequency band. Such results implied that tPBM impacted hemodynamic activities mainly on the endothelial cells of blood vessels at the endogenic oscillation.

### 4.3. Effects of tPBM on Frequency-Specific FC and Global Network Metrics

Instead of evaluating the effects of tPBM on FC within a broad frequency range (0–0.2 Hz) of Δ[HbO] as reported in Urquhart et al. (2020b), we calculated FC at three distinct frequency bands: endogenic (0.003–0.02 Hz), neurogenic (0.02–0.04 Hz), and myogenic (0.04–0.15 Hz), followed by comparisons of frequency-specific FC and global network metrics between three different time periods (i.e., pre, during, post) under either active or sham tPBM condition. To the best of our knowledge, this paper is the first one investigating the effects of tPBM on frequency-specific whole-head FC. Statistical analysis revealed distinct effects of tPBM on FC at different frequency bands.

#### 4.3.1. Effects of tPBM on Endogenic Oscillation of Δ[HbO]

The observed significant enhancement in endogenic FC may be the results of changes in cerebral blood flow (CBF) and metabolism in the microvessels induced by tPBM (Stefanovska et al., 1999). Several existing studies observed an increase in CBF (Uozumi et al., 2010; Poyton and Hendrickson, 2016; Hipskind et al., 2018) and metabolism (Mintzopoulos et al., 2017; Salehpour et al., 2018) after tPBM. Prior studies suggested that an increase in the nitric oxide (NO) level induced by tPBM is responsible for the improved CBF (Lohr et al., 2009; Lee et al., 2017; Keszler et al., 2018). Up-to-date research proved that PBM could be considered an exogenous stimulus that provoked an increase in NO production (Rizzi et al., 2018; Barolet et al., 2021). NO, one of the vasoactive substances produced by the endothelium, significantly influences vascular tone (Mongan et al., 2013). NO is able to trigger vasodilation by activating guanylate cyclase to form cyclic guanine monophosphate (cGMP), which activates protein kinase G (PKG), resulting in decreased levels of Ca^2+^ concentration. The latter prevents myosin light-chain kinase from phosphorylating the myosin molecule, leading to the relaxation of the smooth muscle cells of blood vessels and lymphatic vessels (Charriaut-Marlangue et al., 2013; Mongan et al., 2013). This vasodilation improves cerebral circulation and oxygenation, leading to enhanced FC after tPBM compared to pre-stimulation.

Specifically, the post-stimulation period exhibited boosted FC at the endogenic frequency between rPFC and most other regions of interest (ROIs) (Figures 6A, 7C). FP and DLPFC are associated with working memory, attention, and executive functions, while PMC, M1/S1, and Wernicke are related to motor control and speech fluency. The tPBM-evoked enhancements of FC in these ROIs support prior studies reporting improved executive function (Berman et al., 2017; Blanco et al., 2017), reaction time (Barrett and Gonzalez-Lima, 2013; Grover et al., 2017; Vargas et al., 2017), attention (Disner et al., 2016; Hwang et al., 2016), and working memory (Barrett and Gonzalez-Lima, 2013; Berman et al., 2017).

Furthermore, the strong modulation ability of tPBM on endogenic oscillation within cerebral hemodynamic ISO across the human whole head seen in this study is highly consistent with and well supported by another just-published article of our group (Wang et al., 2022). The report was based on dual-channel, bbNIRS measurements taken from a completely different group of participants and performed by different operators. Similarly, tPBM was reported to significantly enhance the spectral amplitude of Δ[HbO] in the endogenic band by 13% near the tPBM site compared to the sham condition (Wang et al., 2022). The excellent agreement in observing tPBM-evoked strong modulation in endogenic oscillation between two independent studies strengthens the underlying physiological expectation that tPBM facilitates (either directly or indirectly) an increase in NO, which will trigger alterations in endogenic oscillations and then vasodilation within a period of 1–4 min.

#### 4.3.2. Effects of tPBM on Myogenic Oscillation of Δ[HbO]

We also observed significant improvement in myogenic FC following active tPBM (Figures 6B, 7E,F). Oscillations in this frequency band reflect the intrinsic activity of the vascular smooth muscle in response to changes in intravascular pressure (Rowley et al., 2007). The vascular smooth muscle may relax or contract in response to a decrease or increase of vascular pressure. In fact, the myogenic tone is strongly influenced by the release of vasoactive substances produced by endothelium (Mongan et al., 2013). As mentioned above, tPBM evokes an increase of NO, which activates guanylate cyclase to form cyclic guanine monophosphate (cGMP), leading to vasodilation (Charriaut-Marlangue et al., 2013; Mongan et al., 2013). Similar to the endogenic frequency band, the Post-Pre pair of the myogenic frequency band exhibited significant improvement in FC among those ROIs involved closely in working memory, attention, executive functions, and motor control. Moreover, the observation that tPBM enabled to strongly modulate myogenic oscillation within cerebral hemodynamic ISO across the human whole head is also highly consistent with our recent article (Wang et al., 2022). Accordingly, tPBM significantly enhanced the spectral amplitude of Δ[HbO] in the myogenic bands by 23% near the tPBM site compared to the sham condition (Wang et al., 2022).

**Figure 9:**
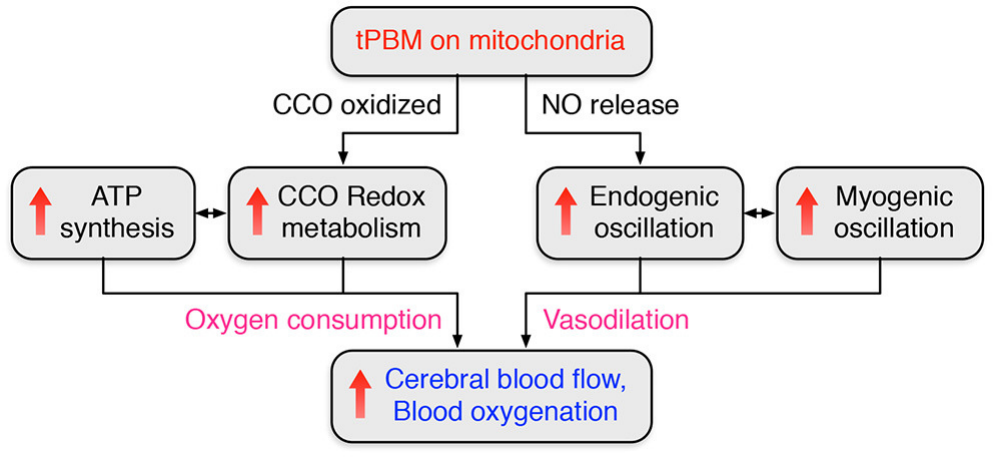
Flowchart to summarize two metabolic-primary hemodynamic events induced by tPBM.

Putting all the knowledge and recent findings together, our observations could be explained or interpreted as follows: tPBM started a cascade process by (1) first triggering the release of NO (besides photo-oxidation of CCO), (2) causing increases in endogenic oscillation and FC, (3) giving rise to the relaxation of the vascular smooth muscle, and thus (4) resulting in changes in myogenic oscillations as well as increases in myogenic FC. This cascade activity takes time in a few minutes for any significant change to be identifiable by physiological or objective measures, as we documented in our recent publications (Wang et al., 2019, 2022). Figure 9 summarizes two metabolic-primary hemodynamic events induced by tPBM. On the one hand, photo-oxidation of CCO enhances CCO redox metabolism and ATP synthesis, leading to a significant increase of Δ[HbO], as observed in Sections 3.1 and 3.2 and our previous studies (Wang et al., 2017b; Pruitt et al., 2020). On the other hand, tPBM activates the release of NO (Rizzi et al., 2018; Barolet et al., 2021), which results in changes in endogenic and myogenic oscillations. Vasodilation caused by the increase of NO leads to an improvement of cerebral blood flow (Lohr et al., 2009; Lee et al., 2017; Keszler et al., 2018).

Furthermore, GTA also revealed tPBM-evoked significant improvements in characteristic path length (*L*_*p*_), global (*E*_*g*_), and local efficiency (*E*_*loc*_) metrics for the myogenic frequency band during the stimulation period (Figure 8). The characteristic path length, defined as the average of the shortest path lengths connecting each vertex to all other vertices, indicates how easily information is transported over the entire network (Stam and Reijneveld, 2007). Meanwhile, global efficiency measures the overall ability to transfer and process integrative information among channels or brain regions, and local efficiency indicates how efficiently the nodes communicate to others if one node is removed (He and Evans, 2010; Rubinov and Sporns, 2010). Thus, shortened path lengths during the active tPBM session indicate that tPBM enabled to increase the transmitting speed for network information exchange. In the meantime, both global and local efficiencies of the topological network were improved perhaps partially because of shortened characteristic path length induced by tPBM.

#### 4.3.3. Effects of tPBM on Neurogenic Oscillation of Δ[HbO]

For the neurogenic oscillation, paired *t*-tests with FDR correction reported that no significant difference was found in any FC line/edge between Post-Pre comparison or between Post-Stim comparison in either channel-wise or region-wise analysis. This observation implied that tPBM had little effect to alter or perturb the FC at neurogenic frequency range. A possible cause of this insignificant effect of tPBM on neurogenic oscillation is that neurogenic fluctuations reflect activities in large arteries and are independent of the endothelium (Stefanovska, 1999; Stefanovska et al., 1999). Accordingly, we can expect that tPBM would have minimal impact on this ISO component if the NIR light stimulated or modulated mainly the endogenic oscillation via NO release, one of the biological products by tPBM.

### 4.4. Limitations and Future Work

We identified two key weaknesses and proposed future work to overcome them.

#### 4.4.1. Understanding Inter-individual Variability vs. Inter-experimental Variability

In Section 3.3, we compared changes in functional connectivity in two cases: (1) over time (pre vs. during vs. post) within each experimental session (active tPBM or sham) and (2) between experimental sessions (tPBM vs. sham). In case (1), the comparison was based on a single experiment setup with the main effect of active tPBM or sham over time, where the data deviation resulted from the inter-individual variability. On the other hand, in case (2), the comparison was made between two experimental sessions, in which the experimental setups of the same subject were not perfectly the same. Thus, in this case, the data deviation resulted from both inter-individual and inter-experimental variabilities (Kreutz and Timmer, 2009). Our results demonstrated clearly that inter-individual variability within one single experimental session (active tPBM or sham) was small enough to obtain significant effects of tPBM on endogenic and myogenic FC. On the other hand, the experimental variability between the tPBM and sham experimental sessions seemed too large, which shadowed the significance of tPBM between the two sessions. The experimental variability could be attributed to the complex setup of the whole-head fNIRS across subjects, which led to larger measurement inconsistency between the two sessions. To address this issue, we may reduce the number of channels and mark the probe locations with a 3D digitizer for improving the LABNIRS measurement reproducibility in future studies.

#### 4.4.2. Potential Contamination of Extracranial Layers to the Interpreted Signals

It is known that fNIRS signals measured on the scalp of human subjects include contributions from the extracranial layers (i.e., scalp and skull). To remove such potential contamination, extra optical channels of fNIRS with a short source-detector (S-D) separation (commonly ~0.8–1.2 cm) have been used for systemic noise removal in task-evoked hemodynamic studies (Zhang et al., 2007; Tian et al., 2011; Yücel et al., 2015; Zhou et al., 2020; Noah et al., 2021), where a cortical region is functionally stimulated by a given task. However, most fNIRS-derived FC studies did not consider this confound effect until a recent report demonstrated that resting-state FC analysis with short S-D correction provides better accuracy than without correction (Paranawithana et al., 2022).

We knowledge another weakness of this study is that the potential contribution from the extracranial layers to the hemodynamic results has not been considered. Under the tPBM scenario, both the superficial and cortical layers of tissue received optical stimulation. Thus, the conventional methods to remove superficial-layer effects are not appropriate in this study. However, even with room for improvement of accuracy, our reported results and conclusions in this study are in good agreement with a recent fMRI-driven report, showing that increases in brain-wide FC of the human brain were observed with connections involving the stimulated hemisphere having a significantly larger increase than those in the contralateral hemisphere (Dmochowski et al., 2020). Several independent studies also confirmed that tPBM enables to stimulate electrophysiological oscillation powers and enhance functional connectivity based on whole-head electroencephalogram (Berman et al., 2017; Zomorrodi et al., 2019; Ghaderi et al., 2021).

In the field of non-invasive neuromodulation, this is the first mechanistic investigation with novel results on the tPBM-induced enhancement on frequency-specific hemodynamic power and FC in healthy humans across the whole head, which are in good agreement with other publications. In future studies, we plan to develop appropriate experimental setups and algorithms that will enable us to remove the confounding factor and thus to confirm/refine the results reported here.

## 5. Conclusions

In this study, we utilized a whole-head fNIRS system concurrently with 1,064-nm tPBM delivered on the right prefrontal cortex to investigate the neurophysiological and/or hemodynamic responses to the light stimulation. First, we implemented the cluster-based permutation test on the tPBM-evoked, whole-head Δ[HbO] signals within the broad frequency range (0–0.2 Hz), which facilitated cortical mapping of cerebral regions of significant increases in Δ[HbO] signals over the right frontopolar area near the tPBM site, confirming the findings more rigorously. Next, we focused more on the intrinsic ISO components of the cerebral activity and analyzed different ISO-dependent metrics, including (1) Δ[HbO] spectral power, (2) frequency-specific FC, and (3) frequency-specific global network metrics. Experimental results revealed that ISO components responded differently to tPBM. Briefly, tPBM significantly increased endogenic Δ[HbO] power across the entire cortical region and enhanced topographical FC between the frontal stimulation site and the central as well as parietal regions. Furthermore, tPBM improved not only the myogenic FC across frontal-parietal cortical regions significantly but also several global network metrics substantially. Such strong effects of tPBM on both endogenic and myogenic hemodynamics may be attributed to tPBM-evoked NO release that would stimulate endogenic oscillations and vasodilation of blood vessels. These findings were consistent with the results reported recently and met the expectation that myogenic oscillation is highly associated with the endothelial activity. Finally, we proved our hypothesis of this study that tPBM would enhance FC at specific frequency bands (i.e., endogenic and myogenic oscillations), resulting from mitochondrial absorption of light given by tPBM.

## Data Availability Statement

The data presented in this study are available upon request, the request can be directed to the corresponding author.

## Ethics Statement

The experimental protocol was approved by the Institutional Review Board (IRB) of the University of Texas at Arlington (IRB # 2017-0859). The patients/participants provided their written informed consent to participate in this study.

## Author Contributions

NT analyzed the data, interpreted the results, and prepared the manuscript. XW assisted data analysis, discussed the results, and reviewed the manuscript. HW recruited the human participants, collected the experimental data, and managed and organized the data. HL initiated and supervised the study, discussed and interpreted the results, as well as reviewed, and revised the manuscript. All authors contributed to the article and approved the submitted version.

## Funding

This research was funded in part by the National Institute of Mental Health, NIH (RF1MH114285).

## Conflict of Interest

The authors declare that the research was conducted in the absence of any commercial or financial relationships that could be construed as a potential conflict of interest.

## Publisher's Note

All claims expressed in this article are solely those of the authors and do not necessarily represent those of their affiliated organizations, or those of the publisher, the editors and the reviewers. Any product that may be evaluated in this article, or claim that may be made by its manufacturer, is not guaranteed or endorsed by the publisher.
